# Integrating breast tumour homologous recombination deficiency status to aid germline *BRCA1* and *BRCA2* variant classification

**DOI:** 10.1016/j.ebiom.2026.106199

**Published:** 2026-03-07

**Authors:** Cristina Fortuno, Jia Zhang, Lambros T. Koufariotis, Georgina Hollway, Scott Wood, John V. Pearson, Peter T. Simpson, Sunil R. Lakhani, Amy E. McCart Reed, Heather Thorne, G Bruce Mann, Anita R. Skandarajah, Lisa Devereux, Qihong Zhao, Dilanka L. De Silva, Geoffrey J. Lindeman, Paul Waring, Paul A. James, Ian Campbell, Amanda B. Spurdle, Nicola Waddell

**Affiliations:** a QIMR Berghofer Medical Research Institute, Brisbane, QLD, Australia; b UQ Centre for Clinical Research, Faculty of Health, Medicine and Behavioural Sciences, University of Queensland, Brisbane, QLD, Australia; c School of Biomedical Sciences, Faculty of Health, Medicine and Behavioural Sciences, University of Queensland, Brisbane, QLD, Australia; d Pathology Queensland, Royal Brisbane and Women's Hospital, Brisbane, QLD, Australia; e Sir Peter MacCallum Department of Oncology, The University of Melbourne, Melbourne, VIC, Australia; f Peter MacCallum Cancer Centre, Melbourne, VIC, Australia; g The University of Melbourne, Melbourne, VIC, Australia; h The Royal Melbourne Hospital, Melbourne, VIC, Australia; i Parkville Familial Cancer Centre, Peter MacCallum Cancer Centre and Royal Melbourne Hospital, Melbourne, VIC, Australia; j The Walter and Eliza Hall Institute of Medical Research, Melbourne, VIC, Australia; k LifeStrands Genomics Australia, Melbourne, VIC, Australia

**Keywords:** Homologous recombination deficiency (HRD), *BRCA1*, *BRCA2*, Tumour mutation signatures, CHORD, Variant classification

## Abstract

**Background:**

Pathogenic germline variants in certain genes are associated with somatic tumour mutation signatures. The use of somatic tumour mutation data has the potential to improve the identification of true pathogenic variants but remains underexplored. We investigated the integration of tumour homologous recombination (HR) deficiency status as a predictor of pathogenicity for germline *BRCA1* and *BRCA2* variants, building on the established link between HR deficiency and germline pathogenic variants in these genes.

**Methods:**

We analysed breast tumour whole-genome sequence and matching germline data from 350 patients across four datasets: Familial Breast Cancer (N = 77), The Cancer Genome Atlas (TCGA-BRCA, N = 96), the MAGIC study (N = 136), and Q-IMPROvE (N = 41). A total of 15,156 germline variants (including structural variations) in *BRCA1*, *BRCA2*, and other cancer genes (*ATM*, *BARD1*, *BRIP1*, *CHEK2*, *PALB2*, *PTEN*, *RAD51C*, *RAD51D, TP53*) underwent variant curation. Patients were categorised based on germline classification as *BRCA1* positive (N = 27), *BRCA2* positive (N = 21), and *BRCA1/2* negative (N = 232), excluding those with *BRCA1/2* variants of uncertain significance (N = 8) and pathogenic or only uncertain variants in other cancer genes (N = 62). Somatic HR status (deficient or proficient) was predicted using three algorithms: HRDetect, CHORD, and HRDsum. HR-deficient and HR-proficient status were significant predictors of germline *BRCA1/2* pathogenic variant status (positive and negative directions).

**Findings:**

The CHORD algorithm, which estimates *BRCA1* and *BRCA2* subtype specifically, added precision contributing evidence towards pathogenicity for the corresponding gene, reaching pathogenic moderate strength for the relevant gene-subtype. Finally, we assessed CHORD HR predictions for variants of uncertain significance in *BRCA1* and *BRCA2*, and reported their tumour HR status for potential use as additional evidence in variant curation.

**Interpretation:**

Analysis across multiple tumour whole-genome sequencing datasets has shown that HR status prediction algorithms can separate profiles for *BRCA1* and *BRCA2* pathogenic variants and provide further evidence at increased weight to aid in the classification of germline *BRCA1* and *BRCA2* variants. Tumour sequencing offers a promising strategy for reducing the uncertainty in germline variant interpretation.

**Funding:**

This work was funded by the 10.13039/501100001026National Breast Cancer Foundation.

**Research in context**

**Evidence before this study**

Pathogenic germline variants in *BRCA1* and *BRCA2* are strongly associated with homologous recombination deficiency (HRD) in tumours, and prior studies have demonstrated links between tumour-based HRD signatures and germline defects. Multiple algorithms have been developed to predict HRD status from tumour sequencing data (e.g., HRDetect, CHORD, HRDsum), but their comparative performance and real-world utility in germline variant classification, particularly for variants of uncertain significance, remain unclear. A comprehensive evaluation of these tools for use in clinical variant classification is lacking.

**Added value of this study**

This study provides a direct comparison of three tumour-based HRD prediction algorithms across multiple breast tumour whole-genome sequencing datasets, showing that HRD status is a stronger predictor of germline *BRCA1/2* pathogenicity than existing pathology markers. It highlights the unique gene-specific utility of CHORD, which improves discriminatory power for *BRCA1* versus *BRCA2*. Importantly, the study explores the application of tumour HRD predictions as weighted evidence to support pathogenicity of uncertain germline variants in *BRCA1* and *BRCA2*.

**Implications of all the available evidence**

Integrating tumour-derived HRD metrics, particularly CHORD's gene-specific predictions, into germline variant interpretation workflows can reduce uncertainty in *BRCA1* and *BRCA2* germline variant classification, enhancing precision oncology through improved risk assessment, tailored management, early screening, and cascade testing in families.

## Introduction

Homologous recombination deficiency (HRD) impairs a cell's ability to effectively repair DNA double-strand breaks and underlies tumourigenesis in a significant proportion of breast, ovarian, prostate, and pancreatic cancers.^1^ Approximately 18–30% of breast cancers have been reported to exhibit HR deficiency.^2^, ^3^, ^4^ Pathogenic germline variations in the hereditary breast and ovarian cancer susceptibility genes *BRCA1* and *BRCA2*, which are crucial for DNA repair through the HR repair pathway, are strongly associated with HRD at the tumour level.^5^ However, HRD has also been associated to some degree with pathogenic germline variation in other DNA repair genes such as *PALB2*^6^^,^^7^ and *RAD51C*.^8^

Somatic mutation signatures linked to HRD in BRCA-associated tumours^9^, ^10^, ^11^ have driven the development of several tools to predict HR status, including HRDsum,^12^ HRDetect,^4^ CHORD,^13^ HRDCNA,^14^ and HRProfiler.^15^ Increasing evidence indicates that a tumour with HRD, regardless of germline pathogenic variant status of the patient, predicts response to poly (adenosine diphosphate-ribose) polymerase (PARP) inhibitor therapy in patients with ovarian,^16^ prostate^17^ and breast^18^ cancer. As clinical trial evidence accumulates, and subject to support from economic modelling studies, tumour genomic profiling is expected to become central to the clinical management of patients at diagnosis. This represents a timely opportunity to evaluate a different clinical use of HRD to guide the management of cancer patients–the value and feasibility of using breast tumour tissue profiles generated at the time of diagnosis to aid *BRCA1/2* germline variant classification.

Despite the growing understanding of inherited genetic variations in cancer genes, and the establishment of ClinGen Variant Curation Expert Panels to develop gene-specific classification guidelines, the number of variants of uncertain significance (VUS) that are not clinically actionable, remains a substantial clinical challenge. The incorporation of many types of evidence in germline variant classification algorithms improves the classification of individual VUS. The use of tumour data to aid variant classification has already been investigated. For example, observing somatic variants that are commonly found in tumours can serve as evidence towards the pathogenicity of the same variant observed in the germline setting.^19^ While, tumour histopathology features have been used to predict the pathogenicity of germline variants in several genes, including *BRCA1/2*, *TP53* and mismatch repair genes.^20^, ^21^, ^22^, ^23^ In this study, we utilise the known association between HRD and *BRCA1/2*-associated breast cancer to explore the use of tumour HRD status in predicting *BRCA1* and *BRCA2* germline variant pathogenicity.

## Methods

### Ethics

No participants were recruited for this study; the study included data from previously published or approved studies. The Q-IMPROVE was approved by a research ethics committee (HREC/2021/QRBW/73637). The MAGIC study and the kConFab consortium received multisite institutional ethics approval from the Peter MacCallum Cancer Centre Human Research Ethics Committee (19/224, HREC/58844/PMCC-2019 and 25/27, HREC/114617/PMCC, respectively) and Governance approval obtained from each hospital site. Written informed consent had been previously obtained as part of these studies. Access to data and the analysis work in this study was approved by the QIMR Berghofer human ethics research committee within project numbers P2095, P2802 and P3527.

### Cohorts of patients with breast cancer

The analysis included whole-genome sequencing (WGS) data of 350 primary breast tumour and blood DNA (germline) samples from patients within four study cohorts (Supplementary Table S1): A Familial Breast cohort (referred to as Familial Breast) comprising individuals with a personal or family history suggestive of hereditary breast cancer with previous WGS^7^ from the Kathleen Cuningham Foundation Consortium for research into Familial Breast cancer (kConFab, based at the Peter MacCallum Cancer Centre, Melbourne, Australia)^24^; TCGA-BRCA, representing a global multi-institutional cohort of individuals generated through The Cancer Genome Atlas (TCGA), with various clinical, genomic, and molecular characteristics of breast cancer^25^; the MAGIC study^26^ based across several clinical and research institutions in Melbourne, Australia (referred to as MAGIC) which includes women with invasive or high grade in situ breast cancer and unknown germline status; and the Queensland IMplementation of PRecision Oncology in brEast cancer (Q-IMPROvE, based across several clinical and research institutions in Brisbane, Australia) cohort of breast cancer samples that underwent WGS prior to treatment as part of a pilot study to test the value of using WGS in the neoadjuvant setting.^27^ Case and sample de-identified IDs used were the same for the previously published studies (Familial Breast, TCGA-BRCA and Q-IMPROvE), while the identifiers for the MAGIC cohort underwent a two-step de-identification. Sex data (self-reported) was collected by the original studies. All tumour samples consisted of fresh frozen tissue except for the MAGIC cohort, which comprised formalin-fixed paraffin-embedded (FFPE)-derived samples. Samples with less than 20× sequencing coverage in tumour or normal samples were excluded. These datasets were used to call somatic variants in tumours for HR prediction, and germline variants in known breast cancer predisposition genes (*BRCA1*, *BRCA2*, *ATM, BARD1*, *BRIP1*, *CHEK2*, *PALB2*, *PTEN*, *RAD51C*, *RAD51D,* and *TP53*).

### Somatic variant calling

WGS data from tumour and patient-matched germline samples were used to identify somatic single-nucleotide variants (SNVs) and insertions and deletions (indels). Specifically, short-read sequencing reads were aligned to the human genome assembly (GRCh38) using BWA-MEM (v0.7.15).^28^ Somatic SNVs were identified using a dual calling strategy using the intersection of the post-filtered output of qSNP (v114-9838e15)^29^ and GATK (v4.0.4.0).^30^ Short insertions and deletions (1–50 bp) were detected with GATK. Sequence alterations were annotated with qannotate (v172-28ea931) to identify somatic-specific variants and to filter variants located within 6 base pairs of a homopolymer region, and with SnpEff (v4.0e)^31^ for gene consequence.

Somatic copy number aberrations (CNA) were identified using the tool ascatNGS (v4.4.1).^32^ The copy number state of each gene was determined by annotation against known Ensembl genes (version 112). Structural variants (SVs) were determined using qSV (v3.10.0-1160.114.2.el7^33^; https://github.com/AdamaJava/adamajava) using both tumour and germline alignments and subsequent filtering to include high-confidence calls in the analysis.

### Single-base substitution signature analysis

The Fit function of the R package signature.tools.lib (v2.4.5) was used to estimate the number of single-base-substitution COSMIC (v2) signatures based on the somatic SNV catalogues of each tumour sample with 1000 bootstraps. The proportions of each signature were calculated based on the total number of variants.

### Correction of the FFPE mutational signature in the MAGIC cohort

Genomic analysis of DNA extracted from FFPE-derived samples can be problematic, as formalin fixation negatively impacts DNA quality and quantity compared to fresh frozen material. The MAGIC cohort tumour DNA was derived from FFPE samples, therefore FFPEsig (https://github.com/QingliGuo/FFPEsig) was used to correct the FFPE noise signatures from the observed mutational catalogues within these samples. This was performed using the unrepaired mode (without uracil DNA glycosylase).^34^ The corrected profiles were then used in the signature analysis and for the HR prediction.

### Homologous recombination deficiency predictions

The somatic tumour variant profiles were analysed for each individual to predict HR status, classified as deficient (HRD) or proficient (HRP) using three prediction methods, CHORD (v2.03),^13^ HRDetect (v2.4.4),^4^ and HRDsum (no version number, see below).^12^

CHORD is a machine-learning-based classifier. This pan-cancer HR predictor uses a random forest model trained with samples from different cancer types of known HRD and HRP tumour genomes,^13^ enabling to identify HRD-associated mutational signatures from WGS data. CHORD uses the somatic SNVs, indels and SVs as input, and primarily infers HRD from the relative proportions of microhomology-mediated deletions and 1–10 kb duplications. Additionally, CHORD utilises patterns of structural duplications characteristic of *BRCA1* deficiency (1–10 kb) or *BRCA2* deficiency (1–100 kb) to distinguish *BRCA1* subtype HRD from *BRCA2* subtype HRD. A score of >0.5 is considered to represent deficiency.^13^

The HRDetect method uses the mutational signatures to predict *BRCA1/2* deficiency as a surrogate for HR deficiency,^4^ including base substitution signatures 3 and 8, indel patterns, SVs and the copy number-based score HRD loss of heterozygosity (LOH). The HRDetect_pipeline function of signature.tools.lib was used for the HRDetect implementation with 1000 bootstraps and “Breast” specific signatures. A score of >0.7 is considered to represent deficiency.^4^

HRDsum score is a summary score previously used in clinical trials and is based on LOH,^35^ large-scale state transitions^36^ and the number of telomeric allelic imbalances^37^ calculated from the somatic copy number profile.^12^ The HRDsum score was estimated for each sample using the modified scripts of the R package scarHRD (https://github.com/bakeronit/brca_mutational_signatures/blob/main/scripts/hrdsum.R).^38^ A cutoff point of 42 is currently considered an US Food and Drug Administration-approved biomarker to select ovarian cancer patients for PARP inhibition,^39^ which we used to differentiate between deficiency and proficiency in this study.

### Germline variant calling and annotation

The germline sequence data were processed with the GATK best practice workflow to detect germline SNVs and small indels. The nanno module of qannotate (https://github.com/AdamaJava/adamajava) was used to annotate SNVs against dbNSFP (v4.1a),^40^ ClinVar (as at 17/02/2025),^41^ and gnomAD (v3.1.2).^42^ The annotations for the nine breast cancer susceptibility genes (*BRCA1*, *BRCA2*, *ATM, BARD1*, *BRIP1*, *CHEK2*, *PALB2*, *PTEN*, *RAD51C*, *RAD51D,* and *TP53*) were extracted, and unique SNVs and indels were collected across 350 individuals.

SVs in the germline genome were identified using DELLY (v0.7.8) for each individual. SVs overlapping with target genes within a 100 bp window were extracted for classification. A total of eight SVs in the related genes from six individuals, which were classified as pathogenic based on the Variant Effect Predictor consequence predicted null (i.e., frameshift, stop loss, coding sequence variants involving complex rearrangements in clinically relevant exons), were included for analyses as high-confidence germline variants.

### Germline variant curation and individual groupings

All variants were reported and classified in relation to the MANE transcripts, as follows: NM_007294.4 (BRCA1), NM_000059.4 (BRCA2), NM_000051.4 (ATM), NM_000465.4 (BARD1), NM_032043.3 (BRIP1), NM_007194.4 (CHEK2), NM_024675.4 (PALB2), NM_000314.6 (PTEN), NM_058216.3 (RAD51C), NM_002878.4 (RAD51D), and NM_000546.6 (TP53). Germline SNVs and indels were classified using a combination of ClinVar^41^ lookups and summary data review, filtering allele frequency (FAF) ≥0.0001 in the gnomAD database, variant effect, and bioinformatic prediction of variant impact using BayesDel,^42^ maximum SpliceAI delta score,^43^ CADD,^44^ as well as the framework suggested by Villani et al., 2024^45^ for 5′ cis-regulatory region variants. Variants in *BRCA1* and *BRCA2* were additionally classified following the ENIGMA *BRCA1* and *BRCA2* Variant Curation Expert Panel (VCEP) specifications (v1.1.0).^46^ Variants were collapsed into one of three classes: pathogenic/likely pathogenic (P/LP), benign/likely benign (B/LB) or VUS. For practicality, each variant was assigned a category related to its class and whether the variant was within *BRCA1/2* or other cancer genes (*ATM, BARD1*, *BRIP1*, *CHEK2*, *PALB2*, *PTEN*, *RAD51C*, *RAD51D,* and *TP53*). The categories were as follows (Supplementary Table S2): A: P/LP_BRCA, B: P/LP_Other genes (where suspicious VUS were also conservatively included), C: VUS_BRCA, D: VUS_Other genes, E: B/LB_BRCA, and F: B/LB = Other genes. The eight high-confidence pathogenic germline SVs identified using DELLY (three in *BRCA1*, three in *BRCA2*, one in *PALB2*, and one in *RAD51C*) were also included in the relevant germline categories. Based on these categories and their combinations in the same individual, samples were grouped into four different groups for analysis (Supplementary Table S3), which included *BRCA1* and *BRCA2* positive groups (individuals carrying a P/LP variant in *BRCA1* or *BRCA2*, respectively) and a *BRCA1/2* negative group (individuals without a detectable *BRCA1/2* P/LP variant). To avoid the confounding effect of pathogenic variants or VUS in the other cancer genes that may be linked to tumourigenesis, two additional groups were created, termed Excluded (individuals with P/LP or only VUS in other selected cancer genes), and BRCA1/2 VUS (individuals with VUS in *BRCA1* or *BRCA2*, and with no other P/LP variants in any genes); both of these additional groups were excluded from the *BRCA1/2* positive and negative reference groups used for main analyses.

### Statistics

#### Likelihood ratio calculations

Likelihood ratios (LRs) associated with each HR dichotomous status (HRP vs HRD) were calculated as predicted by each tool (HRDetect, CHORD, and HRDsum), using previously used methods^20^; this involved comparison of the proportion of HRD-predicted tumours observed for *BRCA1/2* negative individuals compared to that observed for *BRCA1* positive individuals, and separately for *BRCA2* positive individuals. For CHORD-predicted, the LRs were estimated by stratifying HRD status further into *BRCA1* and *BRCA2* HRD subtypes. A sensitivity analysis was performed including individuals in the Excluded groups. The LRs were used to assign an ACMG/AMP evidence strength category and points using Bayesian conversions.^47^^,^^48^

#### Correlation with histopathology data

Further analyses were performed using R v4.4.1 to identify correlations between HR status and histopathology data. We collected tumour histopathology data comprising histological grade and hormone receptor status (ER, PR, HER2, and the combined triple-negative breast cancer, TNBC). Samples with missing histopathology data for a given variable were excluded from corresponding analysis. To examine the association between tumour pathological measures (grade and hormone receptor status), we performed chi-square tests. Effect size was estimated using Cramér's V to assess the strength of association. Correlations between HR status, as predicted by the different tools, and tumour histopathology markers (grade and hormone receptor status) were analysed using Cramér's V to assess the significance of associations. In addition, for CHORD we compared the BRCA1-type HRD probability scores between TNBC and non-TNBC samples using the Wilcoxon rank-sum tests.

### Role of funders

The funders had no role in study design, data collection, data analyses, interpretation, or writing of the report.

## Results

WGS somatic and paired germline data from 350 patients with breast cancer within four cohorts were processed (Supplementary Table S1). Three of these cohorts (MAGIC, TCGA-BRCA and Q-IMPROVE) were comprised of unselected patients with breast cancer, and the fourth cohort comprised patients with familial cancer (additional details in Methods). The average tumour purity of samples was 0.58 (Familial breast: 0.56, TCGA-BRCA: 0.60, MAGIC: 0.60 and Q-IMPROvE: 0.46) (Supplementary Table S1 and Supplementary Figure S1a). To harmonise the data, all data were re-processed with the same pipeline for somatic variant detection. Samples from the MAGIC cohort yielded fewer somatic variants, with a lower tumour mutation burden (TMB) compared to other cohorts (Supplementary Figure S1b) and fewer somatic SV events (Mann–Whitney U p = 2.2e-16) (Supplementary Table S1, Supplementary Figure S1c), likely due to the impact of FFPE-derived samples on DNA quality and lower read depth in these tumour samples.

### Classification of patients based on *BRCA1/2* and other gene variants

**Fig. 1:**
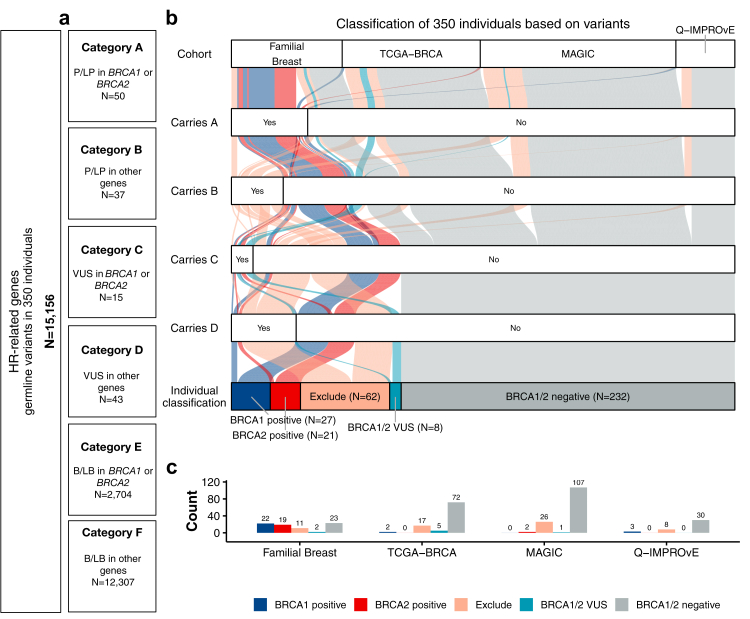
**Germline variant classifications and individual allocation into a germline group based on the presence of germline variants. a)** The total number of germline variants (including SNVs, indels and SVs) identified in the *BRCA1*, *BRCA2*, *ATM*, *BARD1*, *BRIP1*, *CHEK2, PALB2, PTEN, RAD51C*, *RAD51D* and *TP53* genes (N = 15,156). Each variant was classified into six categories (A to F) as defined in the boxes. **b)** The alluvial diagram illustrates the allocation of 350 individuals into five colour-coded germline groups (dark blue: *BRCA1* positive; red: *BRCA2* positive; pink: Exclude; teal: *BRCA1* or *BRCA2* VUS; grey: *BRCA1/2* negative). Individuals were classified into each group based on the four criteria displayed along the y-axis. **c)** The bar plot displays the individuals classified into five groups within each patient cohort (Familial Breast (N = 77), TCGA-BRCA (N = 96), MAGIC (N = 136) and Q-IMPROvE (N = 41)). Abbreviations: B/LB = Benign/likely benign; HR = Homologous recombination, P/LP = Pathogenic/Likely pathogenic; VUS = Variant of uncertain significance.

Across the 350 individuals, a total of 15,156 germline SNVs, indels, and SVs were detected in *BRCA1*, *BRCA2* and non-*BRCA1/2* genes, including HR-related genes (*BARD1*, *BRIP1*, *PALB2, PTEN, RAD51C*, *RAD51D,* and *TP53)* as well as *ATM* and *CHEK2*. These germline variants were grouped into six categories as defined in Fig. 1a and Supplementary Table S2. Each individual person was then placed into a germline group based on the presence of germline variants in the HR genes (Supplementary Table S3). This resulted in 27 individuals being classified as *BRCA1* positive, 21 as *BRCA2* positive, 232 as *BRCA1/2* negative, 62 were excluded from analyses (Excluded) as they were identified to have P/LP or only VUS in other non-*BRCA1/2* genes, and 8 individuals considered for additional analyses (*BRCA1/2* VUS) (Fig. 1b and c).

### HR profile according to cohort and prediction method

**Fig. 2:**
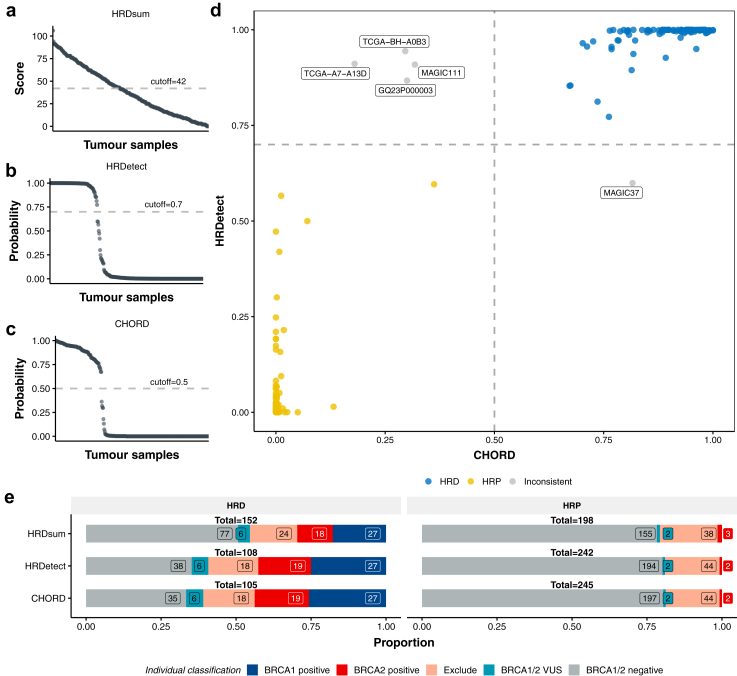
**Comparison of HR status predictions using three approaches.** The distribution of prediction scores or probabilities for all tumour samples using three methods: **(a)** HRDsum, (**b**) HRDetect, and **(c)** CHORD, with cutoff values indicated as grey dashed lines in the plots. **d**) The comparison of HR predictions between CHORD and HRDetect across all samples (N = 350). HRD samples are labelled as blue points and HRP samples as yellow points. Five samples with inconsistent predictions between CHORD and HRDetect are labelled in grey. **e**) The number of samples predicted as HRD or HRP by the three tools indicated on the y-axis. The bars are coloured by the proportion of individuals within each classification group (Dark blue: *BRCA1* positive; red: *BRCA2* positive; pink: Exclude; teal: *BRCA1* or *BRCA2* VUS*;* grey: *BRCA1/2* negative). Abbreviations: HRD = Homologous recombination deficient; HRP = Homologous recombination proficient.

Three methods for HR prediction, based on tumour genomic scars, were used to predict the tumour HR status of the 350 samples. We used the previously published thresholds to determine whether a sample was HRD: CHORD >0.5,^13^ HRDetect >0.7,^4^ and HRDsum ≥42.^12^). HRDsum scores had no clear distinction between HRD and HRP breast tumours irrespective of threshold (Fig. 2a), while the HRDetect and CHORD predicted HRD probabilities showed distinct separation at the selected thresholds for classifying HR status (Fig. 2b and c). The two machine-learning-based approaches, HRDetect and CHORD, were highly concordant with each other and consistently identified 104 HRD and 241 HRP samples (Fig. 2d and Supplementary Figure S2). Only five samples were predicted differently by HRDetect and CHORD. One sample was predicted HRD by CHORD but proficient by HRDetect. This sample was in the Excluded group with a germline P/LP variant in *PALB2* (category B). The other four samples were predicted as HRD by HRDetect but not CHORD: one in the *BRCA1/2* negative group, and three samples in the Excluded group (Fig. 2d, Supplementary Table S4). The non-congruent HR classification for these samples may be attributed to a small number of SV events in one case (MAGIC37, N = 8), or the presence of VUS in other DNA repair genes (*PALB2*, *ATM* or *BRIP1*) Supplementary Table S4).

HRDsum identified more HRD samples than HRDetect and CHORD, predicting 77 samples in the *BRCA1/2* negative group as HRD (Fig. 2e). In comparison, HRDetect and CHORD only predicted 38 and 35 *BRCA1/2* negative samples as HRD, respectively. This suggests that HRDsum may overcall HRD samples, particularly in those non-familial breast cohorts (Supplementary Figure S3). All individuals carrying a pathogenic *BRCA1* germline variant were consistently predicted as HRD by all three methods. However, HRDsum predicted three *BRCA2*-positive individuals as HRP, while HRDetect and CHORD predicted two of them as HRP (Fig. 2e). Interestingly, one of the *BRCA2* positive cases predicted HRP using all tools did not show evidence of a “second hit” in the tumour, as previously reported.^7^ The tumour for the other *BRCA2* positive case predicted HRP contained four copies of the *BRCA2* gene, however the ploidy of this case was 4 suggesting a whole genome duplication event with no specific “second hit” of *BRCA2*. All other individuals with *BRCA1* or *BRCA2* pathogenic germline variants showed evidence of a somatic event leading to loss of the reference allele, or gain of the variant allele (Supplementary Table S4). This suggests that the HR proficient tumour from the two *BRCA2* positive individuals is likely unrelated to the *BRCA2* germline variant, and due to another unknown mechanism of tumourigenesis.

Overall, the proportion of HRD tumours in each of the four cohorts was consistent with the proportion of *BRCA1/2* positive individuals included in that cohort, being highest in Familial Breast where approximately 50% of the individuals carried *BRCA1/2* germline pathogenic variants, and lowest in MAGIC where there was only one *BRCA2* positive individual (Fig. 1c).

The MAGIC cohort consisted of sequence data from FFPE samples, therefore, we tested whether a signature correction for SNVs would alter the HR status prediction. Although there were minor differences in the score assigned, the prediction of HR status did not change after the signature correction for CHORD (Supplementary Figure S4a) or HRDetect (Supplementary Figure S4b).

### Prediction of *BRCA1* and *BRCA2*-associated HRD subtype using CHORD

**Fig. 3:**
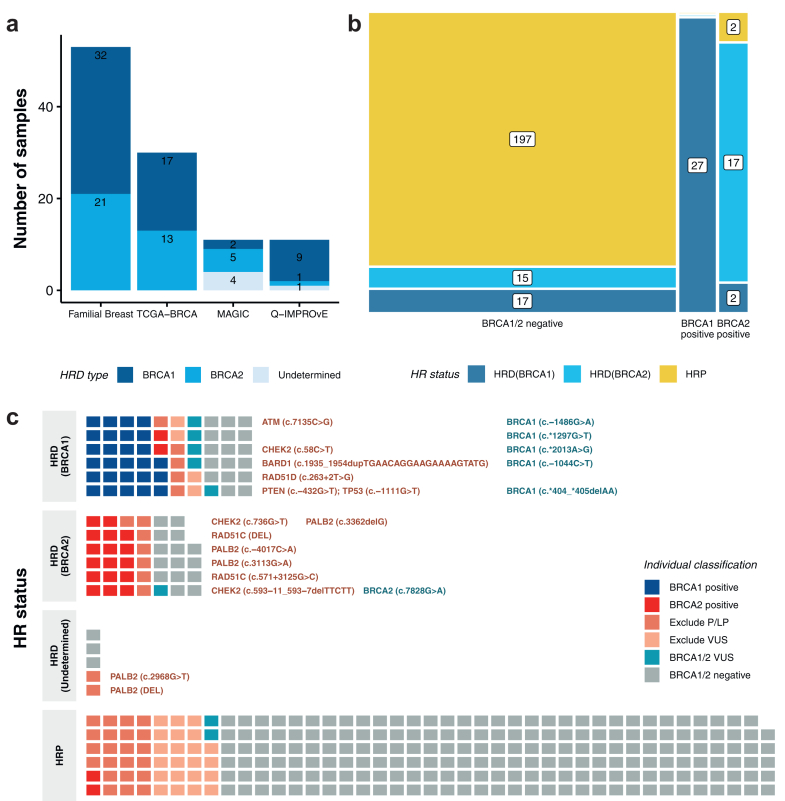
**HRD subtype predicted by CHORD and samples from germline classification groups for different HR statuses. a)** The number of HRD samples differentiated as *BRCA1* and *BRCA2* subtypes or undetermined (five samples). Samples are grouped by the study cohort (Familial Breast, TCGA-BRCA, MAGIC and Q-IMPROvE). **b)** A mosaic diagram shows the proportion of HRD samples in the *BRCA1/2* negative group and *BRCA1* or *BRCA2* positive groups. The colour indicates the HR status predicted by CHORD (Dark blue HRD *BRCA1* subtype, light blue HRD *BRCA2* subtype, yellow HRP). **c)** Waffle plots indicate the number of individuals assigned to each germline group among the samples characterised as tumour HRD (*BRCA1* subtype, *BRCA2* subtype, undetermined) and HRP. The Excluded group is further divided into those harbouring P/LP/suspicious VUS and other VUS in other cancer genes. The P/LP variants detected in non-*BRCA1/2* genes and *BRCA1/2* VUS of HRD samples are labelled in the plot. Abbreviations: HR = Homologous recombination; HRD = Homologous recombination deficient; HRP = Homologous recombination proficient; P/LP = Pathogenic/Likely pathogenic; VUS = Variant of uncertain significance.

In addition to differentiating between HR deficient and proficient status, the CHORD tool can predict whether an HRD case is likely associated with *BRCA1* or *BRCA2* by providing an HRD subtype. CHORD subtype prediction of the 105 HRD samples identified 60 as *BRCA1* type (32 in Familial Breast, 17 in TCGA-BRCA, two in MAGIC, and nine in Q-IMPROvE), and 40 as *BRCA2* type (21 in Familial Breast, 13 in TCGA-BRCA, five in MAGIC, and one in Q-IMPROvE) (Fig. 3a). The remaining HRD samples (four in MAGIC and one in Q-IMPROvE), none falling in the *BRCA1/2* positive germline groups, had an undetermined subtype as they could not be assigned to *BRCA1* or *BRCA2* subtype. While some of these undermined samples may be driven by a small number of somatic SV events detected in these samples, the presence of germline or somatic variation in other genes (e.g., *PALB2* in MAGIC32, MAGIC37) may also contribute to the undetermined subtype categorisation. Further training of CHORD on HRD cases associated with other genes (not *BRCA1* or *BRCA2*) that are associated with HRD (such as *PALB2* or *RAD51C)* may enable CHORD to predict HRD associated with these genes.

All 27 *BRCA1* positive samples were predicted to have a *BRCA1* HRD subtype, and 17 of the 21 *BRCA2* positive samples had a *BRCA2* HRD subtype. For the 232 *BRCA1/2* negative samples, the majority were predicted HRP (n = 197, 85%), with the remainder predicted as HRD with an undetermined subtype (n = 3), *BRCA1* subtype (n = 17), or *BRCA2* subtype (n = 15) (Fig. 3b). A review of the somatic variants for these germline *BRCA1/2* negative samples with *BRCA1* or *BRCA2* HRD subtype predictions revealed relevant somatic changes in these tumours (Supplementary Figure S5 and Supplementary Table S4). The 17 *BRCA1* subtype tumours contained somatic copy number events impacting *BRCA1* (two with loss of one allele, eight with copy neutral LOH or loss, and seven with gains). The copy neutral events are loss of function, as although the tumours have two copies of the *BRCA1* gene, both copies contain P/LP variants indicating there was initially a somatic loss of the ‘wildtype’ *BRCA1* copy then subsequent duplication of the mutant allele. For tumours with a somatic gain of the gene, it is possible that they could harbour promoter methylation of all copies, or undetected variants that impact function of the *BRCA1* gene, for example in distant regulatory elements. The 15 *BRCA2* subtype tumours contained somatic events impacting *BRCA2* (two with homozygous loss, five with loss of one allele, four with copy neutral LOH, three with gains and one with a somatic *BRCA1* splice variant). Of note, there was an additional somatic *BRCA2* splice variant and an inframe *PALB2* variant in these cases (Supplementary Table S4).

Overall, samples predicted to have HRD *BRCA1* or *BRCA2* subtypes by CHORD, were enriched for individuals that were positive for a germline *BRCA1* or *BRCA2* pathogenic variant, respectively, while *BRCA1/2* negative individuals accounted for more than 50% of the HRP samples (Fig. 3c). Interestingly, of the 36 Excluded samples that had P/LP in other cancer genes, 13 had HRD *BRCA1* or *BRCA2* subtype tumours. These variants are specified in Fig. 3c.

### HR status as evidence for or against the pathogenicity of *BRCA1* and *BRCA2* germline variants

To determine if CHORD HR status is a significant predictor of *BRCA1* and *BRCA2* germline variant pathogenicity, the proportion of individuals with each HR status was compared for 232 *BRCA1/2* negative individuals to the 27 *BRCA1* positive or the 21 *BRCA2* positive individuals, in order to calculate LRs towards pathogenicity. For *BRCA1*, observation of an HRP breast tumour corresponded to benign strong evidence for that variant (Table 1), while observation of an HRD breast tumour corresponded to pathogenic moderate evidence. For *BRCA2*, the strength of HRP status as a predictor against pathogenicity was lower than for *BRCA1*, i.e., benign moderate, while the evidence strength towards pathogenicity associated with CHORD-predicted HR status was the same as derived for *BRCA1* i.e., pathogenic moderate.

**Table 1:** LR calculations associated with HR status predicted by CHORD.

*BRCA1*–Dichotomous	*BRCA1/2* negative reference set (n)	%	*BRCA1* positive reference set (n)	%	LR (95% CI)	Evidence strength (Points)
HRP	197	0.85	0	0.00	0.02 (0.00, 0.33)	Benign Strong (−4)
HRD	35	0.15	27	1.00	6.63 (4.88, 9.00)	Pathogenic Moderate (+2)
Total	232		27			

*BRCA1*–Stratified by subtype	*BRCA1/2* negative reference set (n)	%	*BRCA1* positive reference set (n)	%	LR (95% CI)	Evidence strength (Points)
HRP	197	0.85	0	0.00	0.02 (0.00, 0.33)	Benign Strong (−4)
HRD *BRCA1* subtype	17	0.07	27	1.00	23.65 (8.64, 21.57)	Pathogenic Moderate (+2)
HRD *BRCA2* subtype	15	0.06	0	0.00	0.27 (0.02, 4.37)	Not significant (0)
Total	232^a^		27			

*BRCA2*–Dichotomous	*BRCA1/2* negative reference set (n)	%	*BRCA2* positive reference set (n)	%	LR (95% CI)	Evidence strength (Points)
HRP	197	0.85	2	0.10	0.11 (0.03, 0.42)	Benign Moderate (−2)
HRD	35	0.15	19	0.90	6.00 (4.29, 8.39)	Pathogenic Moderate (+2)
Total	232		21			

*BRCA2*–Stratified by subtype	*BRCA1/2* negative reference set (n)	%	*BRCA2* positive reference set (n)	%	LR (95% CI)	Evidence strength (Points)
HRP	197	0.85	2	0.10	0.11 (0.03, 0.42)	Benign Moderate (−2)
HRD *BRCA1* subtype	17	0.07	2	0.10	1.30 (0.32, 5.25)	Indeterminate (0)
HRD *BRCA2* subtype	15	0.06	17	0.80	12.52 (7.36, 21.31)	Pathogenic Moderate (+2)
Total	232^a^		21			

Abbreviations: CI = Confidence interval; HRD = Homologous recombination deficient; HRP = Homologous recombination proficient; LR = Likelihood ratio.

a Includes three tumours with HRD subtype undetermined.

The LR towards pathogenicity increased further for CHORD predictions stratified by the gene-specific HRD subtype (Table 1). Notably, the LR for the HRD subtype inconsistent with the gene being analysed was indeterminate or not significant (based on the confidence intervals).

Similar results were seen when HRDetect was used to predict HR status regardless of subtype (Supplementary Table S5), although the LRs were of slightly lower magnitude in both benign and pathogenic directions using HRDetect (0.02 and 6.11 for *BRCA1*, 0.11 and 5.52 for *BRCA2*) compared to CHORD (0.02 and 6.63 for *BRCA1*, 0.11 and 6.00 for *BRCA2*). HRDsum also resulted in benign strong and moderate evidence for *BRCA1* and *BRCA2*, respectively, but with LRs of the lowest magnitude, while the evidence strength for *BRCA1* and *BRCA2* variants was equivalent only to pathogenic supporting evidence (Supplementary Table S6).

We also conducted a sensitivity analysis for the CHORD subtype-specific predictions considering the additional data for the 62 individuals previously excluded. Among these, two were positive for *BRCA1*, and three were positive for *BRCA2*. Reanalysis considering prediction of *BRCA1* and *BRCA2* positive status irrespective of presence of variants in other cancers genes revealed that LRs did not significantly change and the corresponding evidence categories remained the same (Supplementary Table S7).

### Evaluation of HR predictions in individuals with a *BRCA1/2* germline VUS

There were a total of eight patients with breast cancer carrying *BRCA1* or *BRCA2* germline variants classified as VUS that had been excluded from the reference sets used in the LR analyses. All of the VUS were absent from gnomAD except NM_007294.4(BRCA1):c.-60C > T, with two alleles across gnomAD v2 and v3, and NM_007294.4(BRCA1):c.∗1297G > T with one allele in gnomAD v3.

Six of the VUS (n = 5 *BRCA1* and n = 1 *BRCA2*) identified in six individuals demonstrated tumour HRD (Supplementary Table S4) providing evidence in favour of pathogenicity (Supplementary Table S8). Of the individuals with a *BRCA1* germline VUS with HRD, all had undergone somatic copy number events resulting in bi-allelic loss of *BRCA1* (loss of one allele or copy neutral LOH), providing additional support. Two other VUS in BRCA1 had HRP status providing evidence against pathogenicity (Supplementary Table S8).

### Correlation of predicted HR status with histopathological markers

Breast tumour histological grade, ER status and TNBC status (negative for ER, PR and HER2) were previously shown as predictors of variant pathogenicity for classification of *BRCA1* or *BRCA2* germline variants in analysis of 4477 *BRCA1* pathogenic variant carriers, 2565 *BRCA2* pathogenic variant carriers, and 47,565 breast cancer cases without a known *BRCA1* or *BRCA2* variant.^49^ While number of observations in the dataset analysed for our study is considerably smaller than the previous study,^49^ the overall trends in marker distribution were as expected. For example, based on the histopathology data available in our cohort (Supplementary Table S4), ER-negative status was enriched in *BRCA1* positive individuals (24/27, 89%) compared to *BRCA2* positive individuals (2/21, 10%) and individuals in the *BRCA1/2* negative group (73/232, 31%), which is comparable to trends reported previously for these groups (*BRCA1*, 76%; *BRCA2*, 21%; non-carrier, 23%).^49^ Similarly, the proportion of grade 3 tumours was highest for *BRCA1* positive individuals (19/27, 70%), intermediate for *BRCA2* positive individuals (12/21, 57%), and lowest for those in the *BRCA1/2* negative group (66/232, 28%), comparable to trends reported previously (*BRCA1*, 77%; *BRCA2*, 52%; non-carriers, 33%).^49^

The HR status and histopathological markers for each case, as well as age group and sex were visualised (Supplementary Figure S6a). Using the combined dataset from this study, there was a significant correlation between HRD predicted by all tools and each of the histopathological markers, except for HER2 (Supplementary Figure S6b). For both CHORD and HRDetect, of all pathology markers, correlation was highest for TNBC status (r = 0.49), and lowest for HER2 status (r = 0.16–0.17). Further, there was a significant difference between TNBC and non-TNBC tumours in the distribution of the CHORD-predicted HRD probability (Wilcoxon p < 2.2e-16, Supplementary Figure S6c), and also *BRCA1* subtype predicted probability (Wilcoxon p = 1.7e-08, Supplementary Figure S6d).

## Discussion

Despite the strong known association of tumour HRD with *BRCA1/2*-associated hereditary cancer,^5^ and increasing use of tumour HRD as a biomarker for cancer treatment at the time of cancer diagnosis, this evidence type is not routinely used in germline variant classification of *BRCA1* and *BRCA2* genes. Previous studies have applied CHORD in large cancer genome cohorts and demonstrated its association with HRD and *BRCA1/2*-related repair defects, supporting growing clinical interest in integrating tumour HRD profiling with genetic findings.^13^^,^^50^^,^^51^ However, none of these aforementioned studies performed detailed evaluation of germline *BRCA1/2* pathogenic variants to justify implementation of HRD status as weighted evidence in the *BRCA1/2* specifications. Our study leveraged data from four different cohorts (Familial breast, TCGA-BRCA, MAGIC, and Q-IMPROvE) of breast tumours from 350 individuals. Using HR status predicted with three different algorithms (CHORD, HRDetect, and HRDsum), we estimated the strength of evidence of breast tumour HR profile for predicting pathogenicity of *BRCA1* and *BRCA2* germline variants. We found HR status provides statistical justification for the potential utility of tumour HR profiling as an additional data source for *BRCA1* and *BRCA2* variant classification within existing specifications.^46^ Importantly, since different HR-calling algorithms are used in clinical practice, we investigated and demonstrated differences in the predictive capacity according to the HR testing method. We also assessed whether the prediction methods were robust for application to FFPE-derived samples.

The tumour data from the MAGIC cohort differed to other samples as it was sequenced to a lower read depth and was from FFPE-derived samples. Encouragingly, HR status prediction was unchanged after applying a single base substitution signature noise correction. This suggests that both HRDetect and CHORD prediction methods are robust for application to FFPE-derived samples in our cohort. However, since large-scale genome alterations such as structural and copy-number variations are a feature of HR prediction, we cannot rule out that the lower number of somatic SVs detected in the FFPE-derived tumour material from MAGIC may adversely impact HR prediction for some sample sets. Future studies to benchmark approaches designed to identify HRD using low pass WGS^52^ or compare HRD prediction from matched FFPE and fresh frozen material may provide insight into minimum acceptance criteria for some samples.

A particularly important outcome of this study is demonstrating the practical value of HR status for the assessment of *BRCA1/2* variant pathogenicity within existing guidelines. In the current *BRCA1/2* specifications, clinical data including breast tumour pathology status is currently captured within the ACMG/AMP PP4 and BP5 codes,^46^ with code weights dependent on the combined LR across tumour observations.^53^ To date, the breast tumour biomarkers used routinely in *BRCA1/2* variant classification include grade, ER status and TNBC status.^46^ Calibrations from this previous study showed that most of the predictors were positively and negatively associated with *BRCA1* with supporting evidence strength level, with a few exceptions reaching moderate strength, while all of the predictions associated with *BRCA2* pathogenic germline variant status provided only pathogenic or benign supporting evidence for classification. Results from our study indicate that HR status is overall a stronger predictor of pathogenicity than other tumour pathology features currently used for both genes,^49^ for both pathogenic and benign directions. Evidence strengths applicable to HRD compared to HRP status were generally consistent for CHORD and HRDdetect, with evidence weights higher than when using HRDsum. This highlights the importance of validating HR prediction approaches within testing laboratories. The evidence towards and against pathogenicity associated with CHORD-predicted HRD subtypes reached pathogenic moderate and benign strong strength for *BRCA1* and pathogenic and benign moderate strength for *BRCA2*, demonstrating that HR status is a more useful predictor than previously used tumour markers for variant classification. We also demonstrated that previously used breast tumour pathology markers (grade, ER and TNBC status) are correlated with HR status, although the magnitude of correlation differed by marker and was not complete. Together these observations highlight the importance of avoiding double-counting by not applying multiple correlated sources of evidence derived from the same source. At this point in time, it would be logical to apply the evidence type providing the greatest weight (that is, HRD over e.g., grade), although other pathology markers could still be considered with larger studies confirming the optimal weighting and integration of all markers simultaneously, within the existing PP5 and BP5 clinical codes of the *BRCA1/2* specifications.

Notably, separation by gene-specific HRD subtype predicted by CHORD can add another layer of precision, in that a VUS would not be assigned evidence towards pathogenicity if it was observed to have a tumour HRD profile with the opposite gene (e.g., a VUS in *BRCA2* within a *BRCA1*-like HRD tumour, and vice versa). Each variant was assigned a category related to whether the variant was within *BRCA1/2* or other cancer genes (*ATM, BARD1*, *BRIP1*, *CHEK2*, *PALB2*, *PTEN*, *RAD51C*, *RAD51D,* and *TP53*). This suggests that CHORD may provide more robust evidence towards pathogenicity for the classification of variants in *BRCA1* or *BRCA2* compared to other HR prediction algorithms. The gene-specific HRD subtype from CHORD invokes the exciting possibility that in the future, CHORD can be trained to predict non-*BRCA1/2* subtypes, such as *PALB2* or *RAD51C*. The ability of CHORD to predict which gene is associated with a HR deficient tumour will not only assist with classification of detected variants in these genes, but may also inform genetic analysis for patients with an undiagnosed germline cause of their cancer. For example, we hypothesise a patient with a HR deficient tumour predicted as *BRCA1*-like by CHORD, but with no pathogenic variants identified within the gene, may have variants within regulatory regions or promoter methylation that perturbs *BRCA1* and contributes to the HRD phenotype.^54^ Therefore, inclusion of methylation or gene expression profiling of these individuals may be useful to identify the source of the HR deficiency.

This study also provides evidence weight based on HR predictions for an additional group of eight VUS in *BRCA1/2*, and so may aid future classification of these variants. All except one variant were rare variants located in the 5’ or 3’ UTRs. These variant types are not well captured by existing classification guidelines, and thus additional clinical, functional, and/or predictive data could be beneficial to further inform classification.

Strengths of our work include the use of multiple independent tumour cohorts, application and comparison of three independent HR-calling algorithms, statistical evaluation against curated germline variant classifications, and practical assessment of FFPE compatibility. Limitations include restricted sample size to consider prediction of predict pathogenic variant status for other known HRD-related genes, and lack of promoter methylation data which could influence HR status.

It is worth emphasising the importance of assessing the performance of the HR predictors dataset by dataset, to ensure that the calibration of predictors provides reliable results tailored to the specific characteristics of each dataset. This will include accounting for factors such as differences in HR measurement and/or HR status distribution between different tumour types, and between histological subtypes for a given tumour type. Larger studies will be important to validate our HR-associated LRs in other datasets, including reanalysing the evidence weight associated with HR status by different age groups, clinical and pathology features. In particular, it will be necessary to re-investigate the value of HR status in other tumour types, such as ovarian cancer, for predicting germline variant pathogenicity, since predictive capacity is related to the prevalence of the tumour feature in individuals without a pathogenic germline variant. For example, while up to 80% of *BRCA1* or *BRCA2-*related ovarian cancers present with serous tumour histological subtype, this feature is not predictive of *BRCA1* or *BRCA2* variant pathogenicity since ∼70% of ovarian tumours without *BRCA1* or *BRCA2* pathogenic variants also present with this subtype.^20^

In summary, this study shows that relatively simple calibration approaches can be used to compare and select HR-calling algorithms for use in predicting pathogenicity of germline *BRCA1* and *BRCA2* variants, and that any HR predictor with adequate performance can be used to provide an alternative form of tumour data for application in germline *BRCA1* or *BRCA2* variant classification. This source of evidence could facilitate reclassification of many uncertain variants, enabling patients to receive more appropriate care as hereditary cases, with important implications for treatment decisions and cascade testing of at-risk family members. Importantly, by comparing different tumour HR prediction methods, our results also have relevance for accurate detection of HR status, which is of potential clinical significance as mounting evidence suggests a benefit for the use of PARP inhibitor therapy in HRD breast cancer.

## Contributors

**Cristina Fortuno:** Conceptualisation, Investigation, Formal Analysis, Methodology, Access and verification of underlying data, Writing—Original Draft Preparation. **Jia Zhang:** Investigation, Formal Analysis, Methodology, Visualisation, Data Curation, Access and verification of underlying data, Writing—Original Draft Preparation. **Lambros T Koufariotis:** Formal Analysis, Resources, Writing–Review and Editing. **Georgina Hollway:** Methodology, Writing–Review and Editing. **Scott Wood:** Resources, Writing–Review and Editing. **John V Pearson:** Resources, Supervision, Writing–Review and Editing. **Peter Simpson:** Formal Analysis, Resources, Writing–Review and Editing. **Sunil R Lakhani:** Resources, Writing–Review and Editing. **Amy E McCart Reed:** Resources, Writing–Review and Editing. **Heather Thorne:** Data Curation, Resources, Writing–Review and Editing. **G Bruce Mann:** Resources, Writing–Review and Editing. **Anita R Skandarajah:** Resources, Writing–Review and Editing. **Lisa Devereux:** Resources, Writing–Review and Editing. **Qihong Zhao:** Resources, Writing–Review and Editing. **Dilanka L De Silva:** Resources, Writing–Review and Editing. **Geoffrey J Lindeman:** Resources, Supervision, Writing–Review and Editing. **Paul Waring:** Resources, Writing–Review and Editing. **Paul A James:** Conceptualisation, Resources, Writing–Review and Editing. **Ian Campbell:** Resources, Supervision, Writing–Review and Editing, Funding Acquisition. **Amanda B Spurdle:** Conceptualisation, Supervision, Project Administration, Methodology, Resources, Data Curation, Writing–Review and Editing, Funding Acquisition. **Nicola Waddell:** Conceptualisation, Supervision, Project Administration, Methodology, Resources, Data Curation, Access and verification of underlying data, Writing–Review and Editing, Funding Acquisition. **All authors read and approved the final version of the manuscript.**

## Data sharing statement

The sequence data for the Familial breast cancers was previously deposited in the EGA under accession number EGAD00001004494. The TCGA data was from TCGA-BRCA and accessed from TCGA (https://portal.gdc.cancer.gov). The MAGIC dataset contains de-identified patient information using pseudo-identifiers to protect privacy. Please contact the corresponding authors for data access. The Q-IMPROvE data are available upon reasonable request to the authors.

Scripts used in HRD analysis, germline classification, visualisation are available in GitHub at: https://github.com/bakeronit/brca_mutational_signatures. CHORD is freely available as an R package at the original repository (https://github.com/UMCUGenetics/CHORD).

## Declaration of interests

JVP and NW are co-founders of genomiQa. GH is an employee within genomiQa. The remaining authors declare that there are no competing interests.
